# Supplementary material for: ESR1 NAPA Assay: Development and Analytical Validation of a Highly Sensitive and Specific Blood-Based Assay for the Detection of ESR1 Mutations in Liquid Biopsies
Source: Cancers (Basel). 2021 Feb 1;13(3):556. doi: 10.3390/cancers13030556 (PMC7867152; doi:10.3390/cancers13030556)
Supplement: Supplementary file 1 [file cancers-13-00556-s001.pdf]

SupplementaryMaterials:

ESR1 NAPA Assay: Development and Analytical Validation of a Highly Sensitive and Specific Blood-Based Assay for the Detection of *ESR1* Mutations in Liquid Biopsies

Dimitra Stergiopoulou, Athina Markou, Eleni Tzanikou, Ioannis Ladas, G. Mike Makrigiorgos, Vassilis Georgoulias and Evi Lianidou

Supplementary Table S1. Day to day reproducibility of the *ESR1* NAPA assay using synthetic oligos for each mutation (n = 7).

***ESR1* mutation, (MAF: 9%)**	**Tm (°C)**	**CV%**
Y537S	85.51±0.40	0.47%
Y537C	85.59±0.14	0.16%
Y537N	85.82±0.10	0.12%
D538G	85.58±0.19	0.23%

*: MAF: Mutation Allele Frequency
